# Gadolinium-based layered double hydroxide and graphene oxide nano-carriers for magnetic resonance imaging and drug delivery

**DOI:** 10.1186/s13065-017-0275-3

**Published:** 2017-05-30

**Authors:** Muhammad Sani Usman, Mohd Zobir Hussein, Sharida Fakurazi, Fathinul Fikri Ahmad Saad

**Affiliations:** 1 Materials Synthesis and Characterization Laboratory, Institute of Advanced Technology (ITMA), Universiti Putra Malaysia, 43400 Serdang, Selangor Malaysia; 2 Laboratory of Vaccines and Immunotherapeutics, Institute of Bioscience, Universiti Putra Malaysia, 43400 Serdang, Selangor Malaysia; 3 Department of Human Anatomy, Faculty of Medicine and Health Sciences, Universiti Putra Malaysia, 43400 Serdang, Selangor Malaysia; 4 Centre for Diagnostic and Nuclear Imaging, Faculty of Medicine and Health Sciences, Universiti Putra Malaysia, 43400 Serdang, Selangor Malaysia

**Keywords:** Layered double hydroxides, Graphene oxide, Drug delivery, Gadolinium contrast, Magnetic resonance imaging (MRI)

## Abstract

Gadolinium (Gd)-based contrasts remain one of the most accepted contrast agents for magnetic resonance imaging, which is among the world most recognized noninvasive techniques employed in clinical diagnosis of patients. At ionic state, Gd is considered toxic but less toxic in chelate form. A variety of nano-carriers, including gadolinium oxide (Gd_2_O_3_) nanoparticles have been used by researchers to improve the T1 and T2 contrasts of MR images. Even more recently, a few researchers have tried to incorporate contrast agents simultaneously with therapeutic agents using single nano-carrier for theranostic applications. The benefit of this concept is to deliver the drugs, such as anticancer drugs and at the same time to observe what happens to the cancerous cells. The delivery of both agents occurs concurrently. In addition, the toxicity of the anticancer drugs as well as the contrast agents will be significantly reduced due to the presence of the nano-carriers. The use of graphene oxide (GO) and layered double hydroxides (LDH) as candidates for this purpose is the subject of current research, due to their low toxicity and biocompatibility, which have the capacity to be used in theranostic researches. We review here, some of the key features of LDH and GO for simultaneous drugs and diagnostic agents delivery systems for use in theranostics applications.

## Background

There are various modes of cancer therapy, such as chemotherapy, immunotherapy and radiotherapy. Notwithstanding, the challenge of successful cancer therapy is still existing. Chemotherapy is the most accepted method of cancer therapy amongst the three modes; this is due to availability of various chemotherapeutic agents. However, the major challenge of this method is the chemotherapeutic agents, which do not target the cancerous cells alone but normal cells are also vulnerable to the cytotoxic effects of chemotherapeutic agents [1].

Nanotechnology is a promising field of research, especially in the area of the so-called nanomedicine [2–4]. In recent years, 2D inorganic nanolayers such as layered double hydroxides, graphene and graphene oxide, and metal nanoparticles-based nano-carriers have been used in various drug delivery applications. Their advantages are the reduction in toxicity and improvement of efficacy of chemotherapeutic drugs, which are known to be highly toxic to human cells. Lately, efforts have been made by some researchers to simultaneous dope contrast agents such as gadolinium ion into the aforesaid nano-carries for theranostic applications [5–9].

Layered double hydroxide (LDH) is a class of inorganic nanolayers [10] and one of the most commonly used nano-carriers in drug delivery systems. LDH is an inorganic 2D layered material with interlayer exchangeable anions [11], with the general formula,

$$\left[ {{\text{M}}_{{ 1 {{-}\text{ x}}}}^{{ 2 { + }}} {\text{M}}_{\text{x}}^{{ 3 { + }}} \left( {\text{OH}} \right)_{ 2} } \right]_{\text{x}} { + }\left[ {{\text{A}}^{{{\text{n}} - }} } \right]{\text{ x/n}} \cdot [{\text{mH}}_{ 2} {\text{O]}}$$

where M^2+^ and M^3+^ represent the divalent and trivalent metal cations respectively, and +[A^n−^]x/n represents the interlayer exchangeable anions [11–13]. The inner layers also consist of water molecules which assist in uptake of molecules [14]. LDH synthesis is usually carried out from precursor solutions of the metal salts under alkali pH moderator.

Graphene oxide is a derivative of graphene, a nanomaterial with two dimensions (2D) and an arrangement of sp^2^-bonded carbon atoms. It has stupendous properties such as optical, electronic and thermal stability. GO is formed when the hydrogen atoms are replaced by oxygen atoms during the chemical synthesis. The Hummer’s method is the universally adopted method of GO synthesis from graphite by strong oxidation of the latter [15, 16]. Currently, a lot of effort is been put into exploration of prospective graphene-based materials in biomedical applications, such as nano-carriers for drug and gene delivery, biosensing and bioimaging applications [17].

## Application of LDH in drug delivery

### Drug delivery system

Drug delivery system refers to the typically the use of nano-carriers as *host* to accommodate or load therapeutic agents as *guest* for delivery to specific targets. LDH is one of the most commonly used drug delivery agents. LDH has a 2D-layered structure which gives it a unique ability to intercalate and exchange anions with other materials, which enables it to be used as a drug carrier [18–20]. Another interesting property of LDH is its pH-dependent controlled release properties. This specifically makes its resourceful in pharmaceutical applications. The synthesis of LDH can be conducted via two major chemical routes, which are co-precipitation or ion-exchange method; both methods can be utilized for drug intercalation and have been reported to have high drug loading capacities [21]. Nevertheless, some reports indicate different percentage loading for the methods under the same conditions [22], which may be due to the nature of the therapeutic agents. Co-precipitation is the most adopted technique for LDH synthesis, due to its drug loading ability and is often considered as the easiest method. In co-precipitation method, an aqueous precursor solution of two different metal salts is prepared; to which an anionic guest and alkaline solution are simultaneously added in drop wise manner. The set-up is then kept under stirring at room temperature with continuous hydrogen flush until a pH between 7 and 10 is attained. The mixed solution is then put through aging process for 18 h at 70 °C temperature. The slurry obtained is centrifuged/filtered, washed and oven-dried at 60–80 °C. A variety of anions can be intercalated between the layers, which lead to the formation of multifunctional nanocomposites [23]. The ion exchange technique is much similar to co-precipitation method. However, in ion-exchange method, the guest anion solution is added after the LDH is prepared [22]. As stated earlier, the drug loading capacity of the methods varies based on the nature of the drug or guest anions to be intercalated. Factors such as hydrothermal treatment, aging process, sonication and microwave assisted synthesis have been reported to affect the shapes and other physico-chemical properties of the nanocomposites produced, which in turn influences the drug loading [24].

## Application of GO in drug delivery

The structure of GO consists of sp^3^-hybridized carbons which are composed of different functional groups, such as hydroxyl, carboxyl, and epoxides. The groups are connected to the surface of the GO sheets of the sp^2^ bonding carbon atoms. This enables the efficient loading of aromatic materials such as anticancer drugs onto the sheets [25]. In similarity with LDH drug delivery systems, GO-based drug delivery system is also a representative of a *host*–*guest* interactions in supramolecular chemistry, where the *host* and the *guest* molecules or ions are bonded non-covalently mostly via hydrogen bonds, ionic bonds, van der Waals interactions and hydrophobic bonds [26]. In addition, GO contains a stupendous *π* structure that enables noncovalent *π*–*π* stacking bonding with loaded therapeutics [27]. Due to its composition, GO is equally capable of OH and COOH hydrogen bonding, hydrophobic bonding, embedding and surface absorption [28] with functional groups of various drugs [28]. This facilitates drug to GO bonding for the formation of the nanocomposite and eventually release of the drug in the desired pH [29].

As mentioned earlier, the most commonly used methods of GO synthesis are Hummers’ and Hummers’ modified methods [30], which are top-down chemical approach of synthesizing GO from graphite flakes. Briefly, graphite flakes and sodium nitrate are firstly mixed and concentrated sulphuric acid is then added under constant stirring and allowed to stir for about an hour. Appropriate amount of KMnO_4_ is slowly added to the solution at low temperature. The solution is then allowed to stir further for 12 h at a temperature of 35–50 °C. The solution is then diluted with 500 mL of deionized water. Treatment with 30% H_2_O_2_ is followed. The final suspension is then washed with HCl and H_2_O, filtered and dried at low temperature. GO synthesis is conducted with caution to prevent explosion [31].

## Gadolinium-based nanodelivery system for MR imaging and drug delivery

Magnetic resonance imaging (MRI) is a powerful and one of the most commonly used clinical approaches in diagnosis of cancer patients [32]. It is equipped with high spatial resolution imaging quality with a compact size. It is noninvasive technique and considerably safe for diagnosis [33]. MRI operates under magnetic moments produced from protons in moveable molecules such as water, in a large magnetic field of high magnitude, which are transmitted under radio frequencies as signals to produce images in the MR [34]. The signals generated are of two classes, depending on the needed details of the analysis that is T1 and T2, representing spin–lattice relaxation and spin–spin relaxation mechanisms, respectively. Both signals have their unique colour contrast on different body fluids and tissues [35]. Due to poor sensitivity, MRI often requires the use of contrast agents for better image quality [33]. The contrast agents enhance the signal intensity by increasing the corresponding relaxation rates, 1/*T1* and 1/*T*2, thereby resulting in a bright and dark signals for T1 and T2 respectively, taking lesser times [33, 36].

Gadolinium (Gd) is a rare-earth paramagnetic metal ion which is used in MRI due its ability to interchange freely within a magnetic field. This makes it a useful contrast agent for quality imaging of body organs. Gadolinium and gadolinium chelates [Gd-DTPA (gadopentetate dimeglumine, Magnevist)] are among the first contrast agent approved for use in MRI testing. Gd was introduced as far back as 1988 [37]. Till date, gadolinium-based contrast agents are the only FDA approved contrast agents for MRI to be used on patients with all types of cancer [38] (Table 1).

**Table 1:** FDA approved gadolinium-based contrast agents (GBCAs) for magnetic resonance imaging (MRI)

Brand name	Generic name	Chemical structure
Ablavar	Gadofosveset trisodium [61]	
Dotarem	Gadoterate meglumine [61]	
Eovist	Gadoxetate disodium [61]	
Gadavist	Gadobutrol [61]	
Magnevist	Gadopentetate dimeglumine [62]	
MultiHance	Gadobenate dimeglumine [61]	
Omniscan	Gadodiamide [61]	
OptiMARK	Gadoversetamide injection [61]	
ProHance	Gadoteridol [61]	

Gadolinium chelates are classified into cyclic, which are ligands such as 1,4,7,10-tetraazacyclododecane-1,4,7,10-tetraacetiacid (DOTA) and 1,4,7 tris(carboxymethylaza) cyclododecane-10-azaacetylamide (DO3A) and acyclic ligands which are diethylenetriaminepentaacetic acid (DTPA) and 5,8-bis(carboxymethyl)-11-[2-(methylamino)-2-oxoethyl]-3-oxo-2,5,8,11-tetraazatridecan-13-oic acid (DTPA-BMA). Earlier we have discussed how LDH and GO are used as drug carrier for pharmaceutics. Here we are going to focus on the simultaneous delivery of drugs and imaging agents or the so-called theranostic applications [39].

### Multimodal theranostic drug delivery systems

**Fig. 1:**
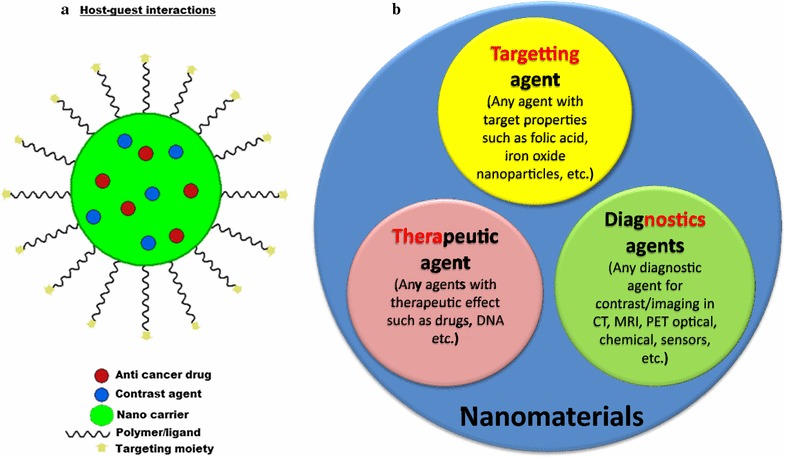
Schematic representation of *host*–*guest* interactions between nanomaterials as carrier serving as *host* and other molecules as *guests* (**a**). Theranostics delivery agent can be obtained by simultaneously loading both the therapeutic and diagnostic agents on the same nanomaterial. On the other hand, multimodal theranostics delivery agent is obtained if all the three agents or more are loaded on a nanomaterial, simultaneously (**b**)

Briefly, theranostics is a newly constructed term derived from the words therapeutic and diagnostic [39], occasionally referred to as theragnostics. It is used in describing the process of simultaneous diagnosis and treatment of diseases, when loaded on a nano-carrier is then referred to as *theranostic drug delivery system* (Fig. 1a, b). However, when only a diagnostic agent is loaded on a nano-carrier, it becomes a *diagnostic delivery system* [40].

Recently, researches have been focused on doping MRI contrast agents-based nanocomposites such as, Gd metal complexes/chelates or Gd metal itself, Gd oxide NPs, iron oxide and other metal nanoparticles, as T1 or T2 relaxation agents, which often involves the use of nano-carriers for delivering the complexes.

*Multimodal theranostic delivery* system refers to delivery system containing more than one diagnostic agents and a therapeutic agent loaded on a nanocarrier with contrast ability for two or more imaging equipments. Multimodal theranostic delivery agent is applied mostly when two or more diagnostic equipments are involved for imaging [41]. As shown in Fig. 1b, nanomaterials as carriers are capable of accommodating various materials based on the final application of the delivery system, from targeting agents, such as forlic acid (FA) to diagnostic theranostic agents for use in MRI, computed tomography (CT), positron emission tomography-computed tomography (PET-CT), sensors and so on, and therapeutics. For instance, Zhang et al. [42] prepared a Gd-based nanocomposite using Si–Ti nanoparticles as the carrier of the Gd and FA as the contrast agent and targeting ligand respectively, for in vivo MRI and near-infrared-responsive photodynamic therapy in cancers. The resulting nanocomposites showed improved T1 weighted MRI contrast. Similarly, Zhang et al. [17] in a bimodal imaging research used GO nano-sheets in the presence of polyethylene glycol (PEG) as a compatibility agent to obtain GO/BaGdF5/PEG nanocomposite. The composite showed promising T1 weighted MR and CT imaging properties. Table 2 depicts previous research works that have reported nanodelivery of contrast agents using various nanoparticles.

**Table 2:** Previous works on gadolinium-based nanoparticles/nanocomposites contrast agents

Carrier	Contrast agent	Remark	Ref	Year
GO	Gd	Improved T1-weighted MRI contrast property	[17]	2015
Si–Ti NPs	Gd	Improved T1-weighted MRI contrast property	[42]	2015
PEG-Gd_2_O_3_	Gd	Gd_2_O_3_ treatment with PEG-silane showed enhanced R_1_ relaxivity	[63]	2007
Gadonanotubes	Gd	Nanotubes showed a R1/pH responsive MRI contrast properties	[64]	2008
Protein-DTPA Gd	Gd	Enhanced T1 particle relaxivities	[65]	2009
Gd-NPs	Gd	Superior contrast properties to commercial contrast agents	[60]	2014
Gd_2_O_3_AuNPs	Gd/Au	Nanoamplifiers showed enhanced contrast	[66]	2013
Gd_2_O_3_NPs	Gd	Intracellular MRI contrast agent	[67]	2011
Gd–Au NCs	Gd/Au	Potential bimodal contrast agent	[68]	2016
Gd–CS DTPA	Gd	In vivo and in vitro results showed enhancement in intensity of MRI signals	[69]	2015

The use of nano-carriers as tools for transporting contrast agents has been a promising start in contrast enhancement research, even more promising is the simultaneous delivery of the contrast agents as well as therapeutic agents using the same nanocarrier (Fig. 1b). Fascinating enough, only a handful of researchers have tried to simultaneously load contrast agents and chemotherapeutic agents onto nano-carriers for drug delivery. To the best of our knowledge, the articles that have reported simultaneous loading of Gd or Gd complexes and chemotherapeutic drugs onto GO and LDH nano-carriers are presented in Table 3.

**Table 3:** Gadolinium based nanocomposites for simultaneous delivery of drug and contrast agents

Carrier	Contrast agent	Active agent	Cell type	Remark	Ref	Year
Mg–Al-LDH	Gd/Au	DOX	Cervical cell (Hela)	Low cytotoxicity in vitro and good CT and T1-weighted MR imaging capabilities	[43]	2013
GO-PEG	Gd	DOX	Human liver cell (HepG2)	Shows greater tumor targeting imaging efficiency	[70]	2012
NGO-PAMAM	Gd	EPI	Glioblastoma (U87)	Inhibit cancer cells growth and good MRI contrast for tumor identification	[56]	2014
GO-DTPA	Gd	DOX	Human liver cell (HepG2)	Improved MRI T1 relaxivity with better cellular MRI contrast and with a substantial cytotoxicity against cancer cells	[55]	2013
Gd(OH)_3_:Mn	Gd	DOX	Breast cancer cell (MDA-MB-231)	High cytotoxicity towards the cancer cells as well promising paramagnetic activity and radiation treatment for cancer	[59]	2016
Fibre	Gd DTPA	Indomethacin	–	Potential theranostic agents	[58]	2016

The synthesis method of LDH and GO plays a role in the loading percentage of the nano-carriers. However, the key factor is the pH of the system, which must be favorable for the *guest* material. For instance, some anticancer drugs are acidic, thus drug loading must done in acidic pH. For cellular uptake, the size and shapes of the nano-carriers play the most significant role.

**Fig. 2:**
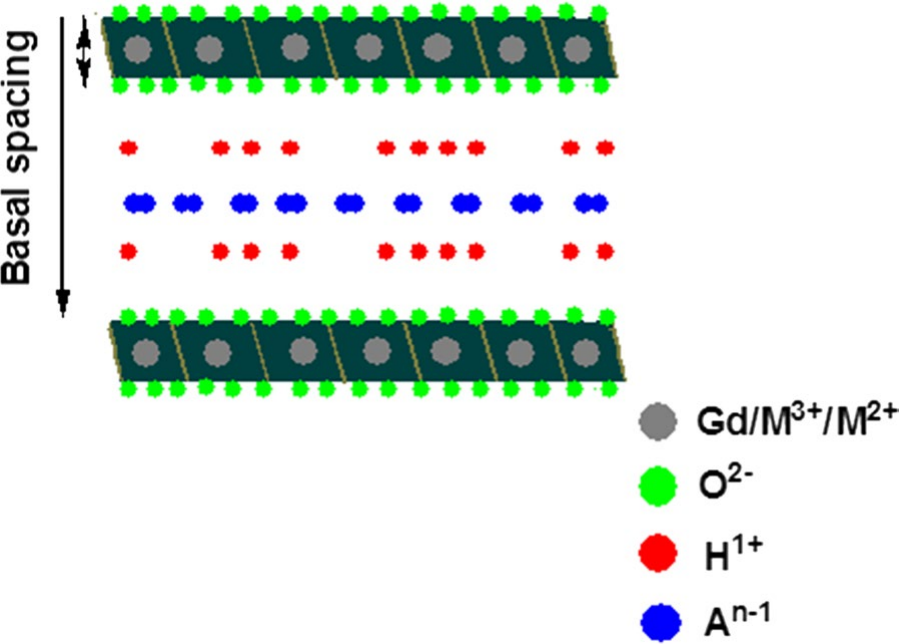
Schematic representation of Gd intercalated within LDH layers

This review is focused on GO- and LDH-based nano-carriers for theranostic applications because of their unique ability to either intercalate or surface-coat other materials in a *host*–*guest* supramolecular interactions. In addition, LDH can accommodate both ionic and non-ionic anticancer drugs [43] at high distribution and sustained release [24]. Figure 2 depicts how theranostic agents can be loaded onto LDH interlayers in the presence of exchangeable anions. It has been reported how nano-carriers such as LDHs have the ability to penetrate cancerous cells [23, 24]. The positively charge outer layers of LDH and the negatively charged cell surface facilitate cell penetration through electrostatic attraction-induced endocytosis and then eventually the anti-cancer drugs are delivered [44, 45]. However, the most reported mechanism of cellular uptake of LDH is via clathrin-mediated pathways [24].

The same mechanism can be applied in contrast agents’ delivery to the cancerous cells; nanocomposite cellular delivery can be observed [46]. In a bimodal imaging theranostic research, simultaneous loading of an anti-cancer drug doxorubicin (DOX), MRI contrast agent, Gd as well as a CT contrast agent, silver nanoparticles (AuNPs) onto MgAl LDH nanocomposites was carried out by Wang et al. [43]. The DOX was coated on the LDH instead of the conventional intercalation of anionic drugs via ion exchange within the LDH interlayers. Interestingly, AuNPs coated on the surface of the LDH-Gd nanocomposites showed much higher CT contrast compared to clinically approved iobitridol contrast agent. Similarly, the in vivo results of the Gd-based LDH composite depicted high T1-weighted MR imaging contrast. Loading of contrast agents into nano-carriers may not only improve the imaging but also reduce the toxicity of the agents themselves, since gadolinium for instance is relatively toxic at certain concentrations [47, 48].

GO-based nanocomposites on the other hand, have been reported to have integrated contrast agents, drugs, nanoparticles as well as other active agents [25, 49, 50]. The high surface area to volume ratio of GO provides opportunity for absorption of metallic materials, drugs and compatibilizers such as polymers, are often used to improve the interaction with other nanoparticles or materials, as stated earlier. Additionally, the thin high surface area 2D structure of GO layers also assists in encapsulating MRI contrast agents such as Gd.

**Fig. 3:**
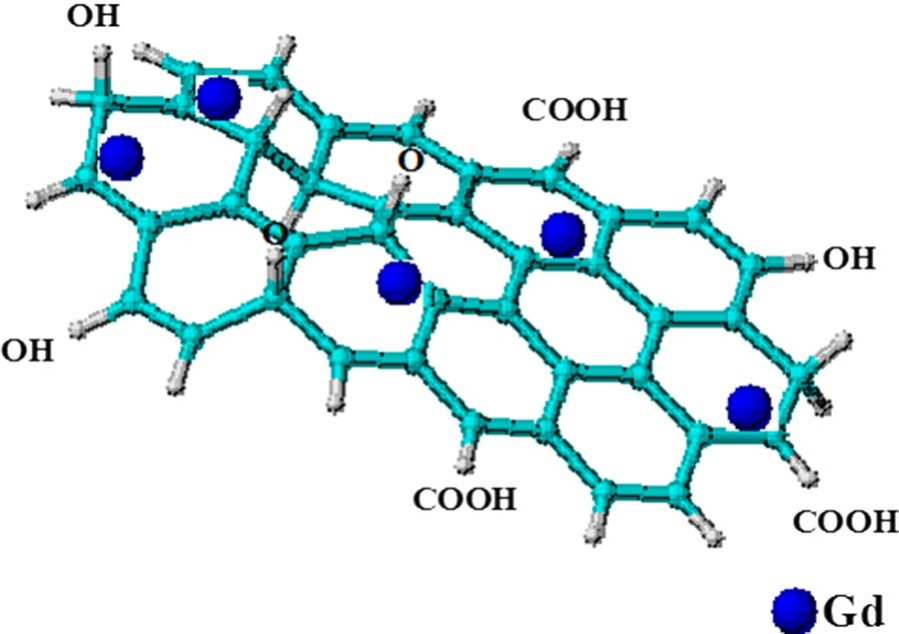
Schematic representation of Gd incorporated onto GO layers

Interestingly, graphene nanomaterial itself is believed to be an anti-cancer in nature [51], however, when incorporated with anticancer agents gives higher therapeutic activity. In similarity with LDH, GO also has the capacity to accommodate both therapeutic agents [50, 52, 53] and MRI/CT contrast agents for theranostic applications at a very low toxicity level [54]. As indicated in the schematic representation in Fig. 3, the Gd is loaded onto the GO sheets via non-covalent *π*–*π* stacking bonding. In the presence of polymers or ligands, hydrogen bonding often occurs depending on the nature of the functional groups of the agent/s involved. A few researchers have incorporated MRI and CT contrast agents into GO for imaging applications (Fig. 3; Table 3), by far only Zhang et al. [55] and Zhang et al. [17] have reported the synthesis of simultaneous delivery of therapeutic agents and loading of contrast agents in GO nanocomposites. In the latter, the GO was functionalized with PEG and transferrin (Tf) ligand for targeting therapy, in the presence of doxorubicin (DOX) as the anticancer agent and Gd as MRI contrast agent for a simultaneous drug delivery and diagnostic research. The GO nanocomposite loaded with Gd expressed an exceptional high quality T1 relative signal intensity as compared to the control used in a 1.5 Tesla (T) medical superconducting MRI system. However, in vivo MRI contrast imaging test was not conducted in their experiment. As for the former, DTPA ligand was employed to chelate the Gd contrast which facilitates bonding with the GO carrier as well as the therapeutic agent. The loaded DOX through physisorption in the simultaneously delivery showed relative low toxicity against human liver cell (HepG2).

In addition, MRI contrast property of the GO nanocomposites was tested using T1-weighted MRI in fluorescence imaging, which interestingly indicated improved contrast against a known commercial Gd contrast agent, Magnevist. Both articles have comparable outcomes, which are improved simultaneous theranostic imaging contrast, high loading and delivery of DOX drug. The synthesis of Gd-based nanographene oxide (NGO) was conducted by Yang et al. [56], who used functionalized NGO as a nanocarrier with Gd for theranostic applications. The gene targeting research was done through poly (amidoamine) dendrimer, which was used in functionalizing the Gd.

The benefit of gene targeting is the ability for the nanocomposite to locate the cancerous cells due to the specificity of the gene targeting agents (Let-7 g miRNAs). More so, the conjugate formed between the anticancer agent Epirubicin (EPI) and the targeting agent improved the theranostic properties of the nanocomposite. Gd-based carriers loaded with anticancer drugs are indeed among the most promising potential tools in fight against cancer, having the advantages of serving as both diagnostic and chemotherapeutic agents. The most important aspect of the nanocomposite is the reduction in the toxicity of the chemotherapeutic agents, which are known to be highly cytotoxic in nature [46, 57]. In a related theranostic research, Zhang et al. [55] conjugated DTPA onto GO nano-carrier in the presence of Gd. DTPA provides platform for the interaction between nano-carriers such as GO and Gd. DOX was used as the therapeutic agent, loaded onto the nanocomposite. The GO-DTPA-Gd/DOX showed improved T1-weighted MRI contrast properties as well as therapeutic properties against HepG2 cells. As clearly indicated in Table 3, only a handful of researches are focused on fabricating the theranostic systems for simultaneous delivery of anticancer drugs and diagnostic agents.

As matter of fact, theranostic systems could be considered the most promising mechanisms for cancer research. A coaxial electrospinning method was utilized by Jin et al. [58] to synthesize core–shell fibers in the presence Eudragit as carrier for delivery of Gd-DTPA as contrast agent. The results showed promising theranostic properties.

Similarly, in a unique approach Yoo et al. [59], used Mn ions to produce the Gd(OH)_3_: MnDOX nanocluster structure in the presence anticancer drug, DOX. The concept is considered promising as indicated by the in vivo toxicity results against breast cancer cells. Gd or Gd-chalets, when incorporated with nanomaterials increase their longitudinal relaxivity through increase of the rotational correlation time. This can be considered as a big advantage for MRI contrasts. Furthermore, Gd-based NPs will enjoy gradual and elongated signal due to the slow release and cellular uptake of the nanoparticles, which subsequently enhances the permeation and retention (EPR) effect. This is conformity with the results obtained by Le Duc et al. [60] in which polysiloxane-encapsulated Gd_2_O_3_ NPs showed perpetual MRI signal in tumor 24 h after injection due to the slow release properties of NPs. Fascinating enough is what these nano-carriers share in common, which is reduction in toxicity of the therapeutics as well as the diagnostics when in the nanocomposite form particularly GO and LDH, which both are capable of drug intercalation. Nonetheless, other NPs do not have such properties.

## Conclusion

Gd-based contrast agents remain the most recognized MRI contrast agent clinically. They are relatively less toxic and easily removed from the body. However, certain factors such as Gd payload, tissue identification, preciseness and other artifacts associated with MRI need to be significantly reduced. Several works on nano-carriers, such as GO and LDH in developing multimodal contrast agents for MRI and CT as well as for simultaneous drug delivery to be used in theranostic applications showed promising results. These novel agents, if developed will help in diagnosis and treatment of terminal diseases, in particular cancer. They may also provide an alternative to the highly toxic chemotherapy, with the use of less toxic nano-carriers in reducing the toxicity of the anticancer agents. This paves way for a new dimension in cancer treatment and management in the near future.

## Abbreviations

Gd: gadolinium
MRI: magnetic resonance imaging
CT: computed tomography
PET-CT: positron emission tomography-computed tomography
NPs: nanoparticles
GO: graphene oxide
NGO: nanographene oxide
LDH: layered double hydroxides
DOTA: 1,4,7,10-tetraazacyclododecane-1,4,7,10-tetraaceticacid
DO3A: tris(carboxymethylaza) cyclododecane-10-azaacetylamide
DTPA: diethylenetriaminepentaacetic acid
DTPA-BMA: 5,8-bis(carboxymethyl)-11-[2-(methylamino)-2-oxoethyl]-3-oxo-2,5,8,11-tetraazatridecan-13-oic acid
NCs: nanocomposites
PAMAM: poly(amidoamine)
PEG: poly(ethylene glycol)
EPI: epirubicin
CS: chitosan
GBCAs: gadolinium-based contrast agents
